# ‘Close confinement tells very much upon a man’: Prison Memoirs, Insanity and the Late Nineteenth- and Early Twentieth-Century Prison

**DOI:** 10.1093/jhmas/jrz027

**Published:** 2019-05-16

**Authors:** Hilary Marland

**Affiliations:** University of Warwick – History, Coventry, West Midlands, United Kingdom

**Keywords:** prison memoirs, insanity, separate confinement, prison discipline, chaplains, doctors

## Abstract

This article explores prisoners’ observations of mental illness in late nineteenth- and early twentieth-century British prisons, recorded in memoirs published following their release. The discipline of separate confinement was lauded for its potential to improve prisoners’ minds, inducing reflection and reform, when it was introduced in the 1840s, but in practice led to high levels of mental breakdown. In order to maintain the integrity of the prison system, the prison authorities played down incidences of insanity, while prison chaplains lauded the beneficent influence of cellular isolation. In contrast, as this article demonstrates, prisoners’ memoirs offer insights into the prevalence of mental illness in prison, and its poor management, as well as inmates’ efforts to manage mental distress. As the prison system became more closed, uniform and penal after the 1860s, the volume of such publications increased. Oscar Wilde’s evocative prison writings have attracted considerable attention, but he was only one of many prison authors criticizing the penal system and decrying the damage it inflicted on the mind. Exploration of prison memoirs, it is argued, enhances our understanding of experiences of mental disorder in the underexplored context of the prison, highlighting the prisoners’ voice, agency and advocacy of reform.

In 1898, prisoner C.3.3, author and playwright Oscar Wilde, evoked the horror of his experiences of prison life, and the despair, anguish and mental breakdown experienced by prisoners in “The Ballad of Reading Gaol”: “And some grow mad, and all grow bad, And none a word may say.”^1^ Sentenced in May 1895 to two years hard labor for homosexual offences, Wilde was removed in November of that year from Wandsworth Prison to Reading, in response to Home Office concerns about his poor health, including his mental state. He was to remain in Reading Gaol until his release in 1897.

Wilde famously attacked the dehumanizing prison system that wore down prisoners and prison staff alike, expressing a keen interest in the plight, sorrows and troubles of the other prisoners, his “pals” as he came to describe them. “Looking at *the others*,” seeing their unhappiness, he claimed, excited his pity, and “broke his obsession with his own fate.”^2^ Composed in Reading Gaol in the early months of 1897, *De Profundis*, Wilde’s lengthy letter to his former lover, Lord Alfred Douglas, described the horror of his prison experiences and revealed his intention of engaging in prison reform.^3^ Following his release, Wilde voiced his concerns about the cruelties of prison life in two letters published in the *Daily Chronicle*, describing how the “terror” of imprisonment was “intensified beyond power of expression by the solitary cellular system of our prisons,” “an example of the cruelty of stupidity.”^4^ He also referred to the large number of men who became insane or weak-minded in prison: “the present prison system seems almost to have for its aim the wrecking and the destruction of the mental faculties. The production of insanity is, if not its object, certainly its result.”^5^ Deprived of books and human interaction, condemned to silence, and “brutalised below the level of any of the brute-creation, the wretched man who is confined in an English prison can hardly escape becoming insane.”^6^ Wilde also asserted that prison doctors had no knowledge of mental disease and were preoccupied with their suspicions that prisoners were feigning insanity. They were “as a class ignorant men. The pathology of the mind is unknown to them. When a man grows insane, they treat him as shamming.”^7^ They were “brutal in manner, coarse in temperament, and utterly indifferent to the health of the prisoners or their comfort.”^8^ The Medical Inspectors of Prisons, meanwhile, did not appear, according to Wilde, to understand the difference between lunacy and idiocy.^9^

Oscar Wilde is certainly unparalleled in the richness and impact of his prison writing. However, as this article demonstrates, many other prison authors were at work in the decades before and after Wilde’s imprisonment, describing the impact of the prison environment and discipline on the bodies and minds of prisoners. As prison regimes shifted from a system that lauded reform and the reclamation of the prisoner in the 1840s and 1850s to one that emphasized uniformity, control and punishment in the 1860s and 1870s, prisoners increasingly recorded their own responses to closed regimes marked by severity and deterrence.^10^ It is their published memoirs that will be the focus of this article.

Dating back to Priestley’s study of 1999, there has been considerable scholarly interest in prison autobiographies, as well as calls to focus on consistent themes within them.^11^ Alyson Brown has shown how prison autobiographies provide rich insights into prisoners’ attempts to survive doing time, gaining a sense of purpose from their imprisonment, while William Forsythe has drawn on prison memoirs to explore how prisoners coped with the loneliness of cellular confinement.^12^ This scholarship, however, has not examined prisoners’ accounts of mental illness and the way it was managed in prisons in any detail. The twenty prison memoirs dating from the late 1860s to the 1920s, drawn on in this article, all reflected, some at length, on the psychological damage resulting from imprisonment. This was regardless of whether these prisoners were held in convict or local prisons, or experienced long- or short-term sentences, and included political prisoners alongside convicted criminals. They related their own struggles and those of other prisoners to resist mental collapse and described the ineffectiveness of the prison in dealing with this. It is suggested that these accounts provide unique insight into mental distress in prison at a point when official reports – seeking to preserve the integrity of prison regimes and discipline – were reluctant to acknowledge that the prison was responsible for the mental breakdown of its inmates.^13^

These writers have had limited exposure compared with Wilde’s contributions and those of well-known prison critics such as Charles Dickens, who lambasted the importation of the system of separate confinement from America to the newly established penitentiaries of England several decades earlier.^14^ Yet these largely middle- or upper-class, well-educated and articulate prison writers generated a robust genre of published material that provides “texts or documents of direct and critical understanding of the discourses and social practices of prison.”^15^ Though some authors wrote anonymously or under *noms de plume*, others were public figures whose trials attracted considerable attention. Susan Willis Fletcher was influential in spiritualist and health reform circles when she was imprisoned for twelve months in 1881 for obtaining goods on false pretences and her trial became a *cause célèbre* with extensive press coverage.^16^ Jabez Spencer Balfour, a large-scale speculator in building schemes imprisoned in 1895 for fourteen years for fraud, had been a Liberal MP and prominent temperance reformer.^17^ The autobiographies of high-profile political prisoners – suffragettes, conscientious objectors imprisoned during the First World War and Irish nationalists – tended to be written with different objects in mind, to promote a political cause or as agendas for prison reform. Yet they too are notable for their detailed observations of mental disorder in prison, observations that contributed powerfully to their cases for reform.

A number of scholars have declared prison writers to be unreliable and biased in their authorship. Written after release and subject to fallibility of memory, it is argued that they corroborated middle-class attitudes towards crime, criminals and punishment and the threat of lower class and criminal “others,” allying themselves to prison officials rather than their fellow inmates.^18^ Frank Lauterbach has argued that such accounts revealed the efforts of the authors to cling to respectability and to dissociate themselves from the average criminal.^19^ While acknowledging the bias in authorship in terms of class, education and background, it can be argued that many prison writers sought to connect to and explore the experiences of other prisoners. Besides, as Forsythe has pointed out, “all writers about Victorian prisons had their own axes to grind,” and official accounts too were strongly biased.^20^ Not all prison writers were well to do; some were career criminals and repeat offenders, who had been in and out of a number of prisons over many years.^21^ Collectively what prison authors wrote – regardless of class, the diversity of writing styles and motives for writing – was notable for the emphases and insights they shared, showing “a consistency of experience that lends credibility to the use of such material for an examination of the impact of the prison on the person.”^22^

In contrast to the narratives of asylum patients, prison writers did not describe themselves as mad, though they often claimed that they came close to madness. Rather they focused on the madness of others as well as the techniques they drew on themselves to avoid falling prey to insanity. Exploration of prisoners’ memoirs thus offers an alternative way of listening to insanity, although often at one remove in describing the mental breakdown of other inmates, and sheds light on the experiences of mental illness in institutional settings other than the asylum that has been far more extensively investigated.^23^ Though the prison was not intended to be a place of containment for the mentally ill, it frequently ended up fulfilling this function, something that has had little acknowledgement in the extensive literature on the confinement of the insane overall.^24^ As with the voices of the “mad” patients examined by Roy Porter, who wrote down their stories in novels, letters and autobiographies, prison authors were eager to shore up their sense of identity, which had been eroded by the machinations of the criminal justice system and the fierce restriction on personal agency imposed by prison discipline.^25^ Prisoners’ memoirs tended to be critical of regimes that caused madness and then failed to do anything to address this. They offer interesting parallels to patients’ accounts, for the most part written by articulate, well-educated and well-off asylum residents, some of which also generated much public attention. They too tended to be highly critical of the institutions where they found themselves or complained of their wrongful confinement.^26^

While Michel Foucault acknowledged the potential impact of prisoners’ accounts, as a “discourse against power,” until recently these accounts were largely ignored in a prison historiography that remained preoccupied with exploring the imposition of penal power in prisons defined by increased observation and rigid discipline.^27^ Yet, through their writing, prisoners demonstrated a form of resistance and a response to prison regimes, which, as Alyson Brown and Emma Clare have argued, capture the intricacies of imprisonment and “the consistency and persistence of certain sentient experiences,” forming “a subjective counter-discourse of the prison.”^28^ Exploration of prison memoirs not only enhances our understanding of experiences of mental disorder in prison, but also adds weight to a growing scholarship that highlights the prisoners’ voice and agency. Their robust and sustained criticisms of the prison system were picked up by prison reformers and taken account of in a number of late nineteenth-century parliamentary enquires.

## Separate Confinement and the Production of Madness in Prison

Pentonville Model Prison in London, taking its first prisoners in 1842, became emblematic of the devastating effects of the regime of separate confinement on prisoners’ minds and a focus of criticism for the anticipated – and later demonstrable – impact on their mental wellbeing. The new system of discipline was roundly criticized by Charles Dickens, while the London *Times* ran a campaign that ensured that what was described, by both supporters and detractors, as Pentonville’s “experiment” and later its failure were played out in a very public manner.^29^ In Pentonville – and subsequently other prisons that implemented separate confinement as the system was rolled out across the country – convicts spent twenty-three hours of each day in silence in their cells, where they worked, ate and slept, being taken out for brief periods to exercise and to attend chapel, where they were seated in separate stalls.^30^ Pentonville’s first prisoners were carefully selected, aged between eighteen and thirty-five, first-time offenders and in good health, fit and able to withstand what was described as a testing regime.

The model for the construction of other prisons in the decades that followed, Pentonville was designed for separation, the prisoners regarded as “defective mechanisms” who could be “remoulded” in a penitentiary devised as “a machine for the social production of guilt.”^31^ The prison housed 520 prisoners in separate cells, with four wings radiating out from a central point, from which all cell doors were visible. Every detail was carefully engineered in terms of cell size and fittings, lighting, heating and ventilation, to ensure that each cell was identical.^32^ Pentonville’s architect, the Surveyor-General of Prisons, Major Joshua Jebb, described how “the individual separation of one prisoner from another is the only basis on which a sound system of prison discipline can be formed,” “depriving a prisoner of the contaminating influences arising from being associated with his fellow prisoners.”^33^ Separate confinement, to be enforced during a period of “probation” prior to transportation, was initially imposed for eighteen months, which it was believed would allow sufficient time for reflection and reform. Under such conditions, which Jebb argued would also give prisoners constant access to “all the good influences” of the chaplains and other prison officers, Pentonville’s advocates argued that prisoners’ mental health would not only be preserved but enhanced.^34^

As the separate system was imposed throughout the British Isles, management of the mind became a major preoccupation of all prison officers: governors, warders, schoolmasters, medical officers and most particularly the chaplains. Despite the avowed benefits of the system, prison staff were enjoined to watch carefully for signs of mental breakdown among the prisoners, and robust surveillance and reporting systems were put in place. The prison chaplains were key figures in efforts to produce deep-seated moral and spiritual reform, through their sermons, the direction of instruction, and, particularly, their communications with prisoners in their cells. They were to assert themselves in matters of the psyche – initially far and above the prison surgeons – claiming that they had the closest oversight and most intimate contact with individual prisoners, and the ability to know, understand and influence their states of mind.^35^

With regard to asylums, Roy Porter compellingly argued that “those who have been shut up have indeed been shut up” or at least no one attended to what they said, except to put down their dislocated speech as a proof of derangement.^36^ In prison, however, the communications of prisoners – whether dislocated speech, accounts of delusions, shouting and screaming, or simply the attempts of prisoners to talk to each other – were closely observed. Prison officers also remained on high alert to detect instances of feigning or shamming insanity. Strictly forbidden to speak to, and thus contaminate, each other, prisoners were, in contrast, enjoined to communicate their deepest feelings to the chaplains, as they established an early and subverted form of talking cure. Separate confinement prompted, Sean Grass has argued, a “prototype of psychoanalysis, complete with divulgences, interpretations, and case histories constructed through the exchanges between analyst and analysand,” chaplain and prisoner “patient.”^37^ The prisoner was trained to think, and sometimes write, in “a confessional, autobiographical, and even self-psychoanalytical way” to produce “narrative coherence, a self-understanding, that would include the origins of criminality, the commission of crime, and – as a final chapter – an awareness of guilt inspired by the beneficient effects of the cell.”^38^ Reverend John Clay, Chaplain of Preston Gaol between 1823 and 1858, described how this form of discipline and his role as “father-confessor” prepared the prisoner for redemption: “a few months in the solitary cell renders a prisoner strangely impressible. The chaplain can then make the brawny navvy cry like a child; he can work on his feelings in almost any way he pleases… and fill his mouth with his own phrases and language.”^39^ Clay thus accentuated the power of the chaplain with his potential for close interaction with prisoners lacking other options for communication and human contact.

In line with his chaplain colleagues in other prisons, John Field at Reading Gaol denied that separate confinement was harmful or productive of mental breakdown; rather it “tends not only to correct and improve the morals but also to strengthen and preserve the mind.” If separate confinement failed, this was related to previous instances of mental breakdown prior to imprisonment, insanity in the family or moral weakness.^40^ At Pentonville, Chaplain Kingsmill anticipated that separate confinement would increase “the better sympathies of human nature” and that acquired knowledge and exercise of the intellectual powers would lead to improvement “of the mind itself.”^41^ His cell visitations, admonishments and instruction, he explained, had produced the self-reflection and repudiation of sin that the reformers hoped for, as prisoners shared “their deepest anxiety and guilt in their isolated cells.”^42^ Tangible proof of the success of his interventions were recorded in his expansive publications, including a letter passed on from a minister in Scotland to Kingsmill, describing how the “seeds of religious instruction which you so faithfully inculcated on my youthful mind, have now begun to take root.”^43^ In 1863 Reverend Charles Gibson, Chaplain to Spike Island Prison in Cork, observed that the separate cell afforded the prisoner “a fine opportunity of looking in upon his own mind, and back upon his past life”; “the prisoner, in his solitary cell, is driven of necessity, to give his confidence to the chaplain, and pour the secrets of his soul into his ear,” underlining the enduring faith of the chaplains in the separate system.^44^

Yet in effect alarming outbreaks of mental disorder were observed shortly after Pentonville opened, a pattern repeated in other prisons as the system of separate confinement was put in place in purpose built and adapted prisons. While Kingsmill would subsequently publish fulsome accounts of his success stories, in his prison journal he noted numerous instances of mental disorder, mania, anxiety, depression, panic, morbid feelings, irritability, delusions, hallucinations, and suicide attempts, and memos describing prisoners’ disturbed behaviour passed between the prison staff on a daily basis.^45^ Officially, just fifteen cases of insanity were recorded in annual reports between 1843 and 1850 at Pentonville. In effect the situation was far worse.^46^ One of Pentonville’s medical officers claimed that the true level of mental disease during the 1840s was five times the reported rate.^47^ Peter Laurie, President of Bethlem Asylum, complained to *The Times* that forty prisoners had been sent from Pentonville to Bethlem by 1847, driven mad by the separate system.^48^ As criticism of Pentonville mounted, efforts were made to tone down the regime of separate confinement, and its duration was reduced, initially to twelve months and then to nine months in 1853.^49^

By the late 1840s Kingsmill was questioning the benefits of separate confinement, initially in his journal and later in published reports, his ideals constantly undermined by the realities of dealing with mentally ill prisoners on a daily basis and by the prisoners’ apparent loss of physical and mental energy under the separate system.^50^ In February 1849 he described convict 1920 as “excited, incoherent & strange in his manner. I am of opinion that his mind is likely to be injuriously affected by the discipline.”^51^ Other prisons officials noted their concerns. Richard Milner, surgeon at Wakefield Prison, asserted in 1847 “cases of mental delusion might be attributed to the separate system, as they were much benefitted by the adoption of the modification of the discipline.” One year later, he observed ten cases of mental delusion, and, with one exception, all recovered after the discipline was relaxed.^52^ Reporting in 1852, Dr Baly, Medical Superintendent at Millbank Prison, observed that separate confinement put the “mental sanity” even of persons of sound mind at risk. Though instruction, intercourse with the prison officers and the promotion of physical health could lessen the risk, the danger remained “since it is a punishment which operates principally and primarily on the mind itself.”^53^

Pentonville’s failure and concerns about the impact of separate confinement in other prisons did not, however, lead to the demise of the system. Far from it, it remained the mainstay of prison discipline and a key feature of the drive for uniformity across the prison estate up until the early twentieth century, even if, in practice, its implementation was flawed and uneven.^54^ The discipline was adapted for local prisons in the 1850s and 1860s, but with many facing conditions of overcrowding and high rates of committal on short sentences, they found it difficult to enforce effectively, and in some prisons a combination of the separate and silent systems – involving silent work in association – was adopted.^55^ Transportation to the colonies ceased during the 1850s, and thereafter the country faced the prospect of punishing its prison population at home, with the period of separate confinement being followed by years of penal servitude. By the 1860s, discouraged by the failure of reformist measures, and persistently high rates of recividism and violent crime, the emphasis of English prison policy had shifted from reform to deterrence, enshrined in the Prisons Acts of 1865 and 1877. After the Prison Commission took over the administration of English and Welsh prisons in 1878, with Edmund Du Cane as the Commission’s chair (1877-95), faith in cellular separation as an efficient, punitive and deterrent system of prison discipline increased.^56^ Separate confinement was still regarded potentially as reformatory but increasingly its value lay in its role as a tool of punishment, and for its emphasis on restricting communication between prisoners and maximizing control.^57^

Under such conditions – the strains imposed by separate confinement itself, combined with the increasing ferocity of penal discipline – incidences of mental illness, despite likely under-reporting, remained high. In 1872 Du Cane suggested that 252 men serving sentences of penal servitude were lunatic or weak-minded, some three per cent of the total of 8,362.^58^ Large numbers of suspected cases of insanity were sent to local prisons, while Dr William Guy, Medical Superintendent at London’s Millbank Prison, where many mentally disturbed prisoners were transferred, reported that some two hundred convicts “unsound in mind… and yet not deemed quite fit for the lunatic asylum” were confined there by 1869.^59^ By the 1860s responsibility for diagnosing, assessing and reporting mental breakdown had passed increasingly to prison medical officers and the role of chaplains had diminished, not least as it was their reformative and spiritual mission that appeared to have failed. Investing a great deal of energy into the detection of feigning, prison doctors typically played down the impact of the prison system on mental health, claiming that prisoners were weak-minded or were already showing indications of mental disturbance when they came to prison.

## Prison Memoirs, the Cell and Mental Breakdown

The final quarter of the nineteenth century was marked by a steady output of prison memoirs, an activity that Anderson and Pratt have related to the closing off of prisons in this period as they became “largely impenetrable to outsiders.” The centralized prison regime, they have argued, hushed debate and silenced dissenting voices within the prison system itself, and “new voices of opposition” emerged in the form of prison authors, articulate and eager to express the need for reform and humanity in the prison system.^60^ Prison authors recorded experiences of both long- and short-term incarceration, though the majority had undergone long sentences of penal servitude in several prisons. Frederick Brocklehurst was confined for only a few weeks in Manchester’s Strangeways Prison, but long enough to comment on the devastating impact of cellular isolation.^61^ Other writers experienced full terms of separate confinement of up to nine months duration (even longer in some cases), followed by years of imprisonment in an associated labor prison. Hard labor produced its own set of mental and physical hardships, particularly distress at not being able to meet daily targets when picking oakum or the dread provoked by the treadwheel, which as one prisoner noted caused even the strongest of men to be led away weeping.^62^

Prisoners’ memoirs were published by a number of mainstream commercial publishers, while serializations or shorter articles (along with book reviews) appeared in general interest periodicals and newspapers, aimed at diverse readerships. These included the conservative and broad circulation journal *The Saturday Review*, the literary periodicals *The Quarterly Review* and *The Athenaeum*, the campaigning *Pall Mall Gazette*, and the Liberal *Daily Chronicle* newspaper. Jabez Spencer Balfour’s *My Prison Life* was first serialized in 1906 in the *Weekly Dispatch*, one of the publishing giant Lord Northcliffe’s newspapers, before appearing in book form.^63^*Five Years’ Penal Servitude*, by One Who Has Endured It, was noted to be a “true account” by *The Saturday Review*, largely based on the credentials of the author and “his education and good behaviour in prison,” and was praised in several other periodicals for its detail and accuracy in describing the prison system.^64^ Given public interest in high-profile trials, such as those of Balfour, Fletcher and Florence Maybrick, imprisoned in 1889 for allegedly poisoning her husband, their subsequent publications were likely to attract a wide readership. Moreover, as Anne Schwan has suggested, publishers appear to have taken the initiative in presenting such texts as contributions to contemporary debates on prison reform and issues such as the plight of “fallen women” and “outcast London,” “for commercial as much as for humanitarian reasons.”^65^

Wilde’s declaration that he had found his salvation through the sympathetic awareness and pity prompted by “looking at the others” in prison was matched by observations on the plight of fellow prisoners in other prison memoirs, and their powerful and evocative accounts are drawn on heavily in this section.^66^ Details of the experiences of other prisoners and sympathy for their position featured in many of these publications. Jabez Spencer Balfour stated his intent of relating the experiences of others, putting himself in the “witness box.”^67^ Describing conditions in Leicester Gaol in the early 1920s, prisoner B.2.15. [R.A. Castle] noted the mental distress of several of his fellow prisoners, explaining that many found it particularly hard to adapt to prison life, such as “one who cried every day for a fortnight.”^68^ Suffragette Constance Lytton employed her prison recollections to promote her cause and to highlight the plight of ordinary women prisoners, an ambition shared by Annie Cobden Sanderson in the unpublished diary she kept while confined in Holloway in 1906.^69^ Florence Maybrick, imprisoned for fifteen years between 1889 and 1904 in Liverpool, Woking and Aylesbury prisons, explained how taking an interest in her fellow prisoners relieved her own suffering.^70^ Susan Willis Fletcher, confined in 1881 in Westminster Prison (Tothill Fields), referred to herself as a “spectator,” observing the penal routine “much as if I had been sitting in a theatre.”^71^ Despite this somewhat detached comment, her eagerness to stress her wrongful confinement and respectability, and her preoccupation with “constructing an exculpatory narrative for spiritualism” that took up much of her book, she remarked on the distress of the other prisoners, speaking for those she believed unable to speak for themselves and urging reform of the prison system.^72^ “When I began to look about me, I… soon came to think of others as well as myself… forming an entire new world, of which I had hitherto no idea.”^73^ She also anticipated the emphasis of the suffragettes in urging women’s involvement in social reform, suggesting that women prisoners were victims of what men did and neglected to do.^74^

Not surprisingly, a number of prison writers focused on their “wrongful confinement,” shedding light on what they perceived as miscarriages of justice, or regretting their own ill-judged actions that had brought them to such a dreadful predicament. Others accepted their wrongdoing and punishment but were disgusted by the manner in which it was carried out in prison. In contrast to the narratives sculpted by prison chaplains to legitimize the regime of separate confinement and to authenticate its achievements, these memoirs were penned chiefly with the objective of providing insights into the failure of prison regimes. They provided searing criticism not only of the uselessness of the prison as a place of reform and rehabilitation, but also of the privations of prison life: the revolting and inadequate diet, the lack of exercise and fresh air, the poor standards of medical care, the hard labor and dreadful, meaningless tasks imposed on prisoners, the dreary boredom, the iron grip of prison weekends, the wasted lives, the loneliness, desperation and sadness.

Describing the routine of the prison in painstaking detail, these accounts provide insights into institutional life and its impact that official records do not – and would not wish to – convey: the humiliation of being transported in the prison van, the disgrace of exchanging one’s clothing for worn and stained prison attire, the hurried and inadequate medical inspection that took place on admission, the loss of personal authority. Florence Elizabeth Maybrick described the horror of being put into uniform and having her hair cut off: “This act seemed, above all others, to bring me to a sense of my degradation, my utter helplessness.”^75^ “Do not fancy that the prisoner is supplied with luxury,” one prisoner explained in his account of twenty years of penal servitude, “every ounce of food that he eats, every inch of clothing that he wears, the very temperature that his cell is warmed to, the amount of work that he does, are all calculated to a nicety, so that he may endure as much as possible for the longest possible time, on the minimum of creature necessaries.”^76^ Such accounts were intended to invoke sympathy but also to assure readers of the harshness of the prison regime and the humiliating treatment that prisoners were subjected to.

The terror of being moved to the prison cell stands out in these accounts. As outlined by Jebb, supporters of separation and the prison chaplains, the cell was to be a tool of reform as well as punishment; for prisoners it was a thing of horror, and prison memoirs provide numerous and evocative accounts of the experience of being isolated, shut in, the tiny space, the door clanking closed, alone “in his separate hell.”^77^ No one did this more effectively than Florence Maybrick, though Anne Schwan has argued that she was put under pressure by her friends and publisher to present a psychological case study of her prison life.^78^ Maybrick’s death sentence was commuted to fifteen years imprisonment after three weeks in the condemned cell, but when moved from Walton Gaol in Liverpool to Woking Prison she explained that she exchanged one horror for another:

“Oh, don’t put me in there!… I cannot bear it.” For answer the warder took me roughly by the shoulder, gave me a push, and shut the door…. I felt suffocated. It seemed that the walls were drawing nearer and nearer together, and presently the life would be crushed out of me. I sprang to my feet and beat wildly with my hands against the door. “For God’s sake let me out! Let me out!”^79^

“No one can realize the horror of solitary confinement,” she went on, “who has not experienced it. It inflicts upon the prisoner at the commencement of her sentence, when most sensitive to the horrors which prison punishment entails, the voiceless solitude, the hopeless monotony, the long vista of tomorrow, tomorrow, tomorrow stretching before her, all filled with desolation and despair.”^80^ She spent just shy of three hundred days in separate confinement, an “inexpressible torture to mind and body,” “known to produce insanity or nervous breakdown more than any other feature connected with prison discipline.”^81^

Frederick Brocklehurst was confined in Strangeways Prison in 1898 after addressing public meetings on social and labor questions, and declining to pay a fine. Though he only served a sentence of one month’s duration, he was horrified by the conditions in Strangeways, his comments appearing in the *Manchester Evening News* prior to being published as *I was in Prison* in 1898. He condemned the diet of “slow starvation,” the miserable plank bed, the cold, and the way a prisoner was “reduced to the level of a wild animal in a menagerie, pacing his cage, merely existing between meal hours.”^82^ Like many other authors, he referred to the size and physical appearance of the cell itself: “Imagine to yourself, a white-washed cube, 7 feet by 8 by 13, with a barred window of ground glass at one end, and a black-painted iron door at the other, and you can form some idea of the dimensions and appearance of a prison cell.”^83^ Austin Bidwell, sentenced in 1873 to twenty years for fraud, despaired “with blinding force” that “for long years that little box – eight feet six inches in length, seven in height and five feet in width… would be my only home.”^84^ By virtue of her respectability Susan Willis Fletcher was accorded special privileges while imprisoned for twelve months in Westminster Prison. Yet she struggled to cope with the cold and damp, restrictions on bathing, the dreadful food, the monotony, and above all the “dehumanizing” solitary cell.^85^ Another prisoner, commenting on the depression produced by the cell, described the atmosphere that “hung about it like a pall, creating an indescribable feeling of sorrow and despair.”^86^

Prisoners roundly condemned the system of separate confinement as a failure. W.B.N. (aka Lord William Beauchamp Nevill), confined in 1898 to five years penal servitude for fraud, served much of his sentence in Parkhurst Prison. While intended to impress the prisoner with the gravity of his offence against society, “and to bring him to a better state of mind” in many cases, he concluded, it had the opposite result. “The solitude and the hopeless monotony, with nothing to think of but the long years of suffering and disgrace ahead, produces nervous irritation, approaching in some cases to frenzy.”^87^ Jabez Spencer Balfour described his sleeplessness, and, confronted with the idea of that “appalling wall of 3,833 days” in prison, “My fear was that I should be overtaken by madness, and I know from the experience of other intelligent prisoners that this is the first trouble that haunts them all.”^88^ During the course of his sentence, Balfour described the overwhelming monotony that was a feature of the various prisons where he served time, “that leads to suicide and attempts at suicide, and which conduces to every form of insanity.”^89^ Close confinement, reflected the author of *Her Majesty’s Prisons* in 1881, “tells very much upon a man” and was reinforced, particularly for those who had occupied “respectable positions in life,” by mechanical and pointless tasks such as oakum picking. Rather than urging reflection and reform, with so little to look forward to “the time is almost necessarily spent in remorseful meditations that become well-nigh maddening as the days slip by. A large percentage of this class of prisoners are packed off to the lunatic asylum… and a still greater percentage have their intellects more or less affected for the rest of their lives.”^90^ Seven years spent in the silence of the whitewashed prison cell inspired Stanley Scott to combine his reminiscences with those he had collected from other prisoners: “As I write, the horror of those days, just ended, rises as a vivid spectre before my mind…. the living death of convict life.”^91^

It seems that there is nothing in the life of a prison that does not tend towards the unbalancing of some of its inmates minds… monotony seems to lead to imbecility – the monotony of that endless round, with silence and whitewash for its surroundings; inactivity, of which there is far more than is healthy, even among men serving hard labour – this is partly responsible for the appalling sanity scourge in British prisons – and then, above all, solitude when on the borders of insanity. There are haunting visions of the past, utter despondency about the future – utter hopelessness – and day by day the cases of coming mental instability, brought to light in the guise of headaches, sickness, and “queerness” at [sick] parade, herald the transfer of more prisoners from the convict stations to the criminal lunatic asylums.^92^

Again and again, the prisoners’ voices highlighted how far removed prison regimes were from achieving the reform and improvement to the mind hoped for in the early days of separation, referring repeatedly to how the use of discipline and the isolation of the cell resulted in deterioration of the mind and mental capacity.

As well as their own struggles with mental distress, prison authors described the insanity of others in their cells or on the “barmy landing.” These included accounts of the mother who was losing her mind, making toys of oakum for her children to play with; the fifteen-year-old who used a broken medicine bottle to cut his leg and then proceeded to eat the rest of it; the youth who attempted suicide and was put in a strait-waistcoat who was then compelled to eat his food like a cat, lying prone on the ground; “men who had come in as prisoners but who went out as madmen, raving all day and most of the night in their padded cells,” and the boy who was not mad but “soft,” who first howled for hours at a time, then took to moaning, and finally “became perfectly quiet and used to lie day after day stretched on the floor in a half stupefied condition.”^93^ “This state of things went on for about a month,” wrote the anonymous author of *Her Majesty’s Prisons*, before the “unfortunate youth” was transferred to the county lunatic asylum “to live at the expense of the ratepayers for the rest of his life.”^94^ Those “whose brains had broken down under this pernicious system of solitary confinement” were moved to padded cells, another ex-prisoner wrote in 1904, “not fit for nothing more than to babble their sad incoherences to the walls of their cells.” The sentence of one man, moved to an asylum, should, he added, have read: “Fifteen months hard labour, *and* to be driven mad by solitary confinement.”^95^

Journals kept by prison doctors and chaplains, annual reports and the publications of prison medical officers devoted much attention to their efforts to distinguish “real” from pretended insanity, which was largely directed to keeping asylum transfers in check. In contrast, prison memoirs describe what this meant for the prisoners, as they were shunted around the prison for observation, being offered nothing in the way of treatment, until they were finally removed to an asylum. Though a few accounts were more positive about the medical attention they received, by and large prison medical officers were criticized for their ignorance of mental illness, failure to diagnose it properly, and excessive claims that prisoners were shamming. Though prison doctors were striving to establish their credentials as experts in dealing with mental disorder in prison, few had been trained in psychiatry and many complained of their heavy workloads.^96^ The author of *Her Majesty’s Prisons* described the doctor in “X-shire” prison as lax and drunk on duty; another account referred to the visits of the doctor as “a brutal farce.”^97^ Florence Maybrick described the case of a prisoner who languished in the prison infirmary at Aylesbury Prison for several months before being certified insane by three doctors and removed to a criminal lunatic asylum. Meanwhile the other prisoners were “disturbed by her ravings, and their feelings wrought upon by the daily sight of a demented fellow creature.” The case of another prisoner, who according to Maybrick, was “in a morbid and depressed state of mind,” ended in suicide.^98^

Irish nationalist and land reformer Michael Davitt related how several of his companions suffered mental illness. In Millbank Prison, “Thomas Ahearn first showed symptoms of madness, and was put in dark cells and strait-jacket for a ‘test’ to the reality of these symptoms.”^99^ However, Davitt also talked about prisoners “putting on the barmy stick,” as well as inflicting wounds on themselves or covering themselves in their own filth, in order to shirk labor, to obtain a relaxation of the discipline or extra diet. Malingerers were justly unpopular, Davitt, concluded, as they made the doctor more suspicious about attempts to feign, risking the neglect of the really sick.^100^ The subject of feigning appears to have resulted in some form of shared ground between prison medical officers and prison authors, who reflected negatively on feigning as it drew the attention of prison doctors away from real cases of illness and mental breakdown. Prisoner W.B.N. described Parkhurst Prison as “half a hospital and half a lunatic asylum,” occupied by a large group of prisoners classed as “W.M., weak-minded” or “balmies.”^101^ Many of this group were described as difficult to manage. While most were really of feeble intellect or partially demented, according to W.B.N., some were sane, and played “balmy” in order to avoid work and punishment for bad conduct. It was difficult for doctors, W.B.N. concluded, to assess whether cases were real or shamming, “for many old convicts are such accomplished actors they are able to imitate the peculiarities of idiocy with wonderful correctness, until the habit becomes second nature.”^102^

Meanwhile, prison discipline and the “crude method” used if madness was suspected – constant watching in the observation cell and the straitjacket – were designed, according to prisoner B.2.15, to exacerbate mental breakdown. He concluded that in some cases “a man or boy can be gradually developing into a tragic mental state and pass unnoticed,” despite monthly visits from the chaplain and the hurried weekly inspection of the medical officer.^103^ Prison accounts explained that it was often prisoners themselves who first noticed and then reported indications of insanity, and that by the time the medical staff had concluded that prisoners were indeed insane their condition had become severe. Conscientious objector Fenner Brockway ended up in the infirmary of Walton Prison in Liverpool after having a wisdom tooth removed, and later worked there as an orderly. “The most distressing sight was ‘Rotten Row’ with cells in which mental cases were confined. Many of the doors were replaced with iron bars, the inmates looking like caged animals. They gibbered idiotically, grinning and masturbating. ‘What on earth were they doing in jail?’ I asked.”^104^

## Survival and Reform

As well as reflecting on their fears of being driven insane by the prison regime, its oppressiveness and isolation, prison authors outlined strategies aimed at self-preservation, to survive the conditions and duration of their imprisonment with their minds intact. “It is this sub-conscious struggle between discipline and mind that embitters the first few months of a first offender’s imprisonment,” socialist and conscientious objector E. Williamson Mason asserted: “Once the mind is free the prisoner has worked out his salvation.”^105^ Indeed, Mason, unusually, saw incarceration as an experience that “made” prisoners. “Prison either makes or blasts a man,” he argued, though only if “one has something self-assertive within one that thwarts the ravages of silence and isolation.”^106^ “The first two years of penal servitude are the hardest to bear,” Michael Davitt asserted, and “test the mental endurance” more than the remaining sentence, solitary confinement being “truly a terrible ordeal to undergo at the commencement.” This was particularly so in Millbank Prison in Westminster, where the prisoner was reminded of the noises of daily life on the outside, with the bells of Big Ben “telling the listening inmates of the penitentiary that another fifteen minutes of their sentences have gone by!”^107^ Weekends were particularly monotonous and provoked memories of families, the allotment, wives shopping, “pals enjoying themselves at the football match, and were hardest to bear,” reported prisoner B.2.15.^108^

Some accounts described religious services as a comfort and praised the work of the chaplains. W.B.N. reported how a visit from a priest saved him from a state of nervous irritation and from smashing his cell.^109^ Another prisoner praised his “muscular Christian” chaplain (soon to be replaced by a less outspoken individual), who addressed the prisoners with respect, and preached courage, observing in one sermon: “No wonder some of you commit suicide, no wonder some go mad, in those miserable holes that are called cells.”^110^ Most prisoners, however, ignored the chaplain’s interventions. Oscar Wilde referred to chaplains as “entirely useless.”^111^ During his two-year stay in Pentonville and Dartmoor, a Ticket-of-Leave Man “never knew a chaplain voluntarily enter a prisoner’s cell to talk to him.”^112^

If prisoners were to survive their sentences and keep their sanity, it was largely by drawing on their own resources. Prisoners described how they developed regimes to reduce boredom, and conducted mental or physical exercises to pass the time. Books were a source of sustenance for many. The author of *Five Years' Penal Servitude* declared in 1878 that if the prison authorities took away books “a very large proportion of the convicts… would become insane.”^113^ As the days “dragged their slow length along in snail-creep fashion,” and tormented by insomnia, Irish political prisoner Jeremiah O’Donovan Rossa attempted to count the stitches in the clothes he was making to force out “the thoughts that troubled me during the day.”^114^ “Suicide and lunacy form a very large item in the effect on England’s treatment of her convicts,” he commented, “and I don’t wonder at it.”^115^ Jabez Spencer Balfour described how he systematically set himself the task of thinking about the problem of sanity and insanity to keep his mind occupied. Though he “resolved never for a moment to be without mental or manual occupation… it requires terrible determination to get the brain straight, to avoid lapsing into regret, thence to despair, and thus to lunacy.”^116^

Prisoners also found ways to disrupt the regime of the prison through their voices and their noises. The prison was intended to be a place of silence, where the prison warders crept in their soft felt slippers or “sneaks,” unheard and thus unobservable by the prisoners, even when they themselves were being watched. Breaking the rule of silence or communicating with other prisoners risked harsh punishment, isolation in a dark cell, a beating, or being placed on a punishment diet. Yet prisoners exerted their agency by developing means of communication. Seasoned gaol-birds, according to prisoner B.2.15, learnt to speak so that they were undetectable using a form of ventriloquism, they tapped and spoke through the heating pipes, some even used a deaf and dumb alphabet.^117^ “No home secretary,” declared Susan Fletcher, “can absolutely govern the tongues of five or six hundred women,” who talked through the ventilators, during exercise and singing in chapel.^118^ More disturbingly, the noise, babble and racket caused by the insane, their screams and cell smashings ruptured the quiet at night, and prison memoirs repeatedly comment on being woken from sleep by the terrible shrieks of the mad, “the bestial ravings of some poor wretch who had reached the limits of his endurance.”^119^

Towards the end of her sentence Fletcher spoke of the total despair that overcame her, her own health breaking down: “the cold, the darkness, the horrible character of all my surroundings, the hopeless condition of the constantly changing swarm of bloated, drunken, miserable women, sent to prison for short terms, only to become more hardened and depraved, weighed heavily upon my heart.”^120^ Florence Maybrick claimed to have suffered a nervous breakdown as her sentence drew to a close, a result, she concluded, of hard labor and insomnia. She also described how the silence had a weakening effect on memory and language, as a form of mental dumbness set in that made prison life endurable after many years confinement, and she became closed to the world outside.^121^ The cold, darkness, silence and solitude, Susan Fletcher explained, “are not curative or reformatory or humanising influences. They disease the body and depress, stupefy and debase the mind.”^122^ Many spoke of the loss of memory or vacancy of mind resulting from separate confinement. W.B.N. described how many prisoners “gave way” mentally, being “little better than half-witted” by the time their separate confinement ended.^123^ Frank Brocklehurst described how prisoners became emotionally and mentally inert, as the prison system reduced “its victims to a dead unthinking level.”^124^

In 1922, conscientious objectors Quaker Stephen Hobhouse and socialist Archibald Fenner Brockway produced a detailed and insightful report on English prisons, drawing on their own prison experiences, prisoners’ memoirs, and the recollections of prison staff and inmates. Separate confinement, one of their ex-prisoner informants explained, served “to drive the man more and more into himself… it leads to a brooding which poisons the whole life.”^125^ Hobhouse, who served four months in Wormwood Scrubs and eight in Exeter Gaol in 1916, described “the full pressure” of his experiences of separate confinement by misguided nineteenth-century reformers. He described how prison life seemed designed to destroy a man’s sense of personality and initiative. His insistence on communicating with the other prisoners landed him a further spell in solitary confinement: “That no permanent mental damage made its appearance during this last and hardest phase of my imprisonment, I attribute entirely to the spiritual equipment, with which I was… endowed,” religious faith, the love of his wife and friends, and his sense of justice in his cause, advantages not shared by all prisoners.^126^ He dreaded the “appearance of a spell of dazed vacancy of mind, of indifference to everything; for this seemed to be a prelude to the decay of mind and… a not uncommon result of long imprisonments.”^127^ Canadian-American sociologist Erving Goffman’s work on total institutions, published in 1961, described how like-situated individuals, cut off from wider society for lengthy periods of time, might experience a form of personal extinction as they adapted to institutional regimes. Yet already four decades earlier, Brockway and Hobhouse castigated long-term imprisonment for producing excessively high rates of mental deterioration, as vigorous, self-centred and introspective activities at the start of sentences were followed by the exhaustion of prisoners’ physical and mental resources, deterioration, apathy and torpor.^128^

Though few writers made derogatory comments of the kind expressed by Jabez Spencer Balfour, who referred to his fellow inmates as “scum,” “like animals,” “a different species” with their abnormally protruding “criminal ear,” spelling out the distance between himself and other prisoners, some were frightened of the other prisoners.^129^ One Who Has Endured It, author of *Five Years' Penal Servitude*, was alert to the dangers imposed on the mind by separate confinement, but also dreaded its end, and being “brought into daily, hourly contact with some of the ruffians and blackguards I had hitherto been able to keep at a distance.”^130^ O’Donovan Rossa was disparaging about the other prisoners, but concluded that he preferred their company rather than the solitary cell.^131^ While most prison autobiographies described the “otherness” of their fellow inmates, at the same time they connected with them through sufferings in common or sympathized with the predicament of those less fortunate, even when these shared experiences crossed class lines. Stephen Hobhouse suggested that the main aspects of the system and its impact on character and mentality “seemed to… be of a similar nature for all prisoners involved.”^132^

Florence Maybrick’s account, Anne Schwan has suggested, stressed empathy and shared experiences, underscored by her frequent use of the term “we.”^133^ In Susan Fletcher’s case her prison sentence appears to have strengthened a commitment to health reform and women’s rights, and her book included a chapter entitled “A Plea for Prison-Reform” and an appendix “What Prisons Are, and What They Might Be.”^134^ She described the predicament of “the feeble, the old, the rheumatic, and those debilitated… by intemperate habits” who “have dreadful suffering.” The large numbers of women who were constantly in and out of prison because of drunkenness were “unfortunate rather than guilty,” and in need of reformatory provision rather than imprisonment.^135^ Maybrick offered recommendations for prison reform, criticizing prisons for locking up mentally ill people and for producing mental breakdown, and urged separate provision for the weak-minded.^136^ She appeared to be well informed on levels of insanity in prison, referring in her book to newspaper accounts that estimated that these were seven times the rate for the general population, even after deducting those coming into prison insane. She also commented on the fact that a reduction in the term of separate confinement in 1898 (when a new Prison Act was passed) was reflected in a declining rate of mental illness.^137^

*Five Years’ Penal Servitude* was lauded for familiarizing the reader with the “strange, sad world within prison limits,” and drew attention to defects in the treatment of criminals, advocating shorter sentences for first offenders, and harsher treatment for habitual criminals. Du Cane was questioned about the book and it was referred to in evidence presented to the Kimberley parliamentary commission of 1879.^138^ Michael Davitt provided extensive testimony to the same commission on prison conditions, diet, labor, medical treatment and the cruelty of the warders.^139^ Along with other prison authors, he particularly urged the separation of young and first time offenders from habitual prisoners and railed against the severity of prisons, issues taken up increasingly by critics of Du Cane’s era, including the Howard Association and Humanitarian League.^140^

Prisoners’ accounts were echoed, their views and accusations confirmed, in periodical publications, the press, novels and plays, by social reformers and prominent literary figures. Just as Charles Dickens had protested the introduction of the separate system in the 1840s, authors Charles Reade and John Galsworthy were to condemn its continued usage as a system of punishment into the late nineteenth and early twentieth centuries.^141^ Prison inmates, *The Speaker* magazine argued in 1895, were “the victims of tyranny as merciless as it was stupid and revolting.”^142^ In a letter to *The Nation* in 1909 Galsworthy cited several accounts of the impact of separate confinement on the minds of prisoners, drawn from prisoners themselves and those working with them, underlining the importance of their testimony. One “highly-strung young woman” who had served a long term of penal servitude, reported that

It is like nothing else in the world… no words can paint its miseries, nothing that I can say would give any idea of the horrors of solitary confinement – it maddens one even to think of it. No one who has not been through it can conceive the awful anguish one endures when shut up in a living tomb, thrown back upon yourself.^143^

In evidence to the Gladstone Committee in 1895, which recommended a relaxation of severity and a renewed focus on reformation, Colonel Baker of the Salvation Army, described how many discharged convicts coming under their care, were incapable of pursuing ordinary occupations. “They are mentally weak and wasted, requiring careful treatment for months after they have been received by us.”^144^ A number of ex-prisoners also provided evidence, describing the severity and regimentation of the system, poor standard of prison staff and lack of reformatory approaches.^145^ Ex-prisoner, Mr. E., attributed high levels of suicide to the severity of prison discipline in his evidence, and described men who had become simple-minded and the nerves and strain that imprisonment induced from constantly striving to avoid trouble. “Separates” was terrible, but so to was the shock of moving to hard labor after a period in isolation.^146^

Prison medical officers persisted in playing down the connection between mental illness and prison regimes, but by the 1890s complaints on the failures of prisons emerged increasingly from the prison system itself.^147^ In 1894 Reverend William Morrison, Chaplain at Wandsworth Prison from 1887 to 1898, argued that the severe and highly deterrent regime operating in prisons by the 1890s was responsible for driving prisoners mad and for further debilitating others who were constitutionally weak. In a piece published in *The Fortnightly Review* under the title “Are our Prisons a Failure?” he asserted that rates of insanity in local prisons had doubled between 1875-77 and 1890-92 as penal discipline became harsher, from 113 per 10,000 to 226 per 10,000 compared with 8 per 10,000 in the general population. He acknowledged that many prisoners might be mentally unstable when entering prison, but warned that “our discipline may punish them, may shatter their intellect, may drive them mad, but it will never deter them from pursuing a career of crime.” Recidivism, he suggested, might well be associated with this debilitating process, which made many men unfit to earn their daily bread.^148^

## Conclusion

One line of Oscar Wilde’s “Ballad of Reading Gaol” was perhaps misleading: “And none a word may say.”^149^ Though silenced under the regime of separate confinement, the voices of prison authors were clear, loud and largely united in their criticism of the prison, its regime, and its impact on the mental health of its inmates, and had been for several decades before Wilde himself went to prison. “Conditions must change – they cannot remain the same,” declared prisoner B.2.15. “The time will come when the present day cast-iron system of Solitary Confinement and mental anguish… will be looked upon in the same way as we regard the physical horrors which are now largely a thing of the past in this country.”^150^ By the early twentieth century, when this account was published, the prison system had re-entered an era of penal reformism. According to prison authors, this had yet to affect their experiences of imprisonment, particularly with regard to mental health.

Why did the prisoner write? In part the production of these memoirs appears to have been a way for prisoners to make sense of their experiences after leaving prison and to assert their voice and agency. While we have to be aware that publishers keen to expand their sales might have encouraged authors to emphasize their interest in reform, many urged change to what they regarded as a useless as well as destructive form of punishment, and based their recommendations on their experiences. Wilde and other prison writers acknowledged their debts to other prisoners, as they spoke and wrote for them, and, for some, observing other prisoners quite simply passed the time. No doubt the memoirs were also written to provide an income from audiences fascinated with criminal activities and prison experiences and occupation for those unable to re-enter society and their previous employment. These accounts might have been exaggerated or designed to attract the sympathy of their readers and to sell copy. However, their repetition of the same themes and experiences – and even the same words, the “horror” of separate confinement, the “cage”-like cell, “monotony” and “torture” – suggest their authenticity.

Whether the narrated speeches of the reformed, voices of complaint or protest, or reportage of other people’s experiences, prison narratives provide insights into the particular circumstance of mental breakdown in prison, the prison as a place of ruin of the mind, and also of complex psychological encounters whether they be between chaplain and prisoner or the prisoners’ own efforts to manage their minds and resist mental breakdown. Prison narrators shared many features with the accounts of asylum patients in terms of resistance to their predicaments. While many asylum patients claimed that they had been wrongfully confined and protested against this, prisoners claimed that incarceration in prison itself was wrongful and prisons inappropriate to the task of reform. Prisons, like asylums, were described, by both sets of narrators as likely to produce mental deterioration rather than improve the mental state of their inmates. Some prisoners found a means, as their writing indicates, of mitigating this process, while reporting on the devastating impact of the prison overall on the mind, memory and cognition. Though many memoirs described prisoners’ innermost feelings and individual psychology, they were preoccupied with survival rather than redemption. There is little evidence of the reflection and reform hoped for by the chaplains in the early years of separate confinement expressed in these accounts. Rather they provide evocative, detailed and at times distressing insights into regimes of confinement and day-to-day struggles to survive institutional life and to retain self-worth and a sense of identity.

## Funding

This work was supported by the Wellcome Trust (103341/Z/13/Z).

## Footnotes

1 Oscar Wilde, “The Ballad of Reading Gaol,” 1898, lines 212, 569-570. Reproduced in *Oscar Wilde: The Soul of Man and Prison Writings*, ed. with an Introduction by Isobel Murray (Oxford: Oxford University Press, 1990), 187.

2 Peter Stoneley, “‘Looking at the Others’: Oscar Wilde and the Reading Gaol Archive,” *Journal of Victorian Culture* 19 (2014): 457-480, 457.

3 Oscar Wilde, *De Profundis* (1897; London: Methuen, 1905). *De Profundis* has attracted a large scholarship, including Regenia Gagnier, *Idylls of the Marketplace: Oscar Wilde and the Victorian Public* (Aldershot: Scolar, 1986); Josephine M. Guy, “Wilde’s *De Profundis* and Book History: Mute Manuscripts,” *English Literature in Transition* 55 (2012): 419-440.

4 To the Editor of the Daily Chronicle, 27 May [1897]; To the Editor of the Daily Chronicle, 23 March [1898], Wilde’s first and second post-prison letters to the *Daily Chronicle*. Reproduced in *Oscar Wilde*, 159-168, 190-196. See also *The Letters of Oscar Wilde*, ed. R.H. Hart-Davis (New York: Harcourt, Brace & World, 1963) and Martyn Housden, “Oscar Wilde’s Imprisonment and an Early Idea of ‘Banal Evil’ or Two ‘Wasps’ in the System. How Reverend W.D. Morrison and Oscar Wilde Challenged Penal Policy in Late Victorian England,” https://forhistiur.de/2006-10-housden/

5 To the Editor of the Daily Chronicle, 23 March [1898], 193.

6 Ibid. That the separate system did indeed lead to insanity at Reading Gaol, as Wilde argued, was confirmed by the steady number of removals of prisoners to nearby Moulsford Asylum in the mid-1890s: Stoneley, “Looking at the Others,” 469-470.

7 To the Editor of the Daily Chronicle, 27 May [1897], 167.

8 Ibid., 23 March [1898], 195.

9 Ibid., 27 May [1897], 167.

10 William James Forsythe, *The Reform of Prisoners 1830-1900* (London: Croom Helm, 1987), esp. ch. 6.

11 Philip Priestley, *Victorian Prison Lives: English Prison Biography 1830-1914* (London: Pimlico, 1999); Sarah Anderson and John Pratt, “Prisoner Memoirs and their Role in Prison History,” in *Punishment and Control in Historical Perspective*, ed. Helen Johnston (Houndmills: Palgrave Macmillan, 2008), 179-198; Alyson Brown, *English Society and the Prison: Time, Culture and Politics in the Development of the Modern Prison, 1850-1920* (Woodbridge: Boydell, 2003), ch. 2. Forsythe advocated a thematic approach in Forsythe, *Reform*, 206.

12 Brown, *English Society*; Bill Forsythe, “Loneliness and Cellular Confinement in English Prisons 1878-1921,” *British Journal of Criminolog*y 44 (2004): 759-770.

13 For the harm caused to prisoners in institutions reluctant to acknowledge the impact of the regime on mental wellbeing, see Catherine Cox and Hilary Marland, “Broken Minds and Beaten Bodies: Cultures of Harm and the Management of Mental Illness in Mid- to Late Nineteenth-century English and Irish Prisons,” *Social History of Medicine* 31 (2018): 688-710.

14 For Dickens’s commentary on prisons, see Charles Dickens, *American Notes for General Circulation* (London: Chapman and Hall, 1842); Sean Grass, *The Self in the Cell* (London: Routledge, 2003), esp. ch. 4; David Wilson, “Testing a Civilisation: Charles Dickens on the American Penitentiary System,” *The Howard Journal of Criminal Justice* 48 (2009): 280-296; Helen Johnston, “‘Buried Alive’: Representations of the Separate System in Victorian England,” in *Captured by the Media: Prison Discourse in Popular Culture*, ed. Paul Mason (Cullompton: Willan, 2005), 103-121; Philip Collins, *Dickens and Crime*, 3^rd^ edition (New York: St. Martin’s Press, 1994), esp. chs V and VI.

15 Steve Morgan, “Prison Lives: Critical Issues in Reading Prisoner Autobiography,” *The Howard Journal* 38 (1999): 328-340, 337.

16 Susan Willis Fletcher, *Twelve Months in an English Prison* (Boston: Lee and Shephard and New York: Charles T. Dillingham, 1884). See Anne Schwan, *Convict Voices: Women, Class, and Writing about Prison in Nineteenth-Century England* (Durham, NH: New Hampshire University Press, 2014), ch. 4.

17 Jabez Spencer Balfour, *My Prison Life* (London: Chapman and Hall, 1907); D. Bythell, “Balfour, Jabez Spencer (1843-1916)” (23 September 2004), *Oxford Dictionary of National Biography* (*DNB*).

18 Frank Lauterbach, “‘From the Slums to the Slums’: The Delimitation of Social Identity in Late Victorian Prison Narratives” and Monika Fludernik, “‘Stone Walls Do (Not) a Prison Make’: Rhetorical Strategies and Sentimentalism in the Representation of the Victorian Prison Experience,” in *Captivating Subjects: Writing Confinement, Citizenship, and Nationhood in the Nineteenth Century*, ed. Jason Haslam and Julia M. Wright (Toronto: University of Toronto Press, 2005), 113-143, 144-174. See also Alyson Brown and Emma Clare, “A History of Experience: Exploring Prisoners’ Accounts of Incarceration,” in *The Persistent Prison: Problems, Images and Alternatives*, ed. Clive Emsley (London: Francis Boutle, 2005), 49-73.

19 Lauterbach, “‘From the Slums,’” 120.

20 Forsythe, *Reform*, 207.

21 For example, B.2.15 [R.A. Castle], *Among the Broad-Arrow Men. A Plain Account of English Prison Life* (London: A. and C. Black, 1924); Stanley Scott, *The Human Side of Crook and Convict Life* (London: Hurst & Blackett, 1924).

22 Brown, *English Society*, 17.

23 For asylum patients’ accounts of their experiences of institutional life, see e.g. Allan Beveridge, “Life in the Asylum: Patient’s Letters from Morningside, 1873-1908,” *History of Psychiatry* 9 (1998): 431-469; Michael Barfoot and Allan Beveridge, “Madness at the Crossroads: John Home’s Letters from the Royal Edinburgh Asylum, 1886-87,” *Psychological Medicine* 20 (1990): 263-284; Geoffrey Reaume, *Remembrance of Patients Past: Patient Life at the Toronto Hospital for the Insane, 1870-1940* (New York: Oxford University Press, 2000). Jade Shepherd included patients’ letters in her exploration of Broadmoor Criminal Lunatic Asylum: Jade Shepherd, “‘I am very glad and cheered when I hear the flute’: The Treatment of Criminal Lunatics in Late Victorian Broadmoor,” *Medical History* 60 (2016): 473-491.

24 From the vast literature on the confinement of the insane, see e.g. Andrew Scull, *The Most Solitary of Afflictions: Madness and Society in Britain, 1700-1900* (New Haven, CT: Yale University Press, 1993) and *Insanity, Institutions and Society, 1800-1914*, ed. Joseph Melling and Bill Forsythe (London: Routledge, 1999). Janet Saunders, “Institutionalised Offenders: A Study of the Victorian Institution and its Inmates, with Special Reference to Late Nineteenth-Century Warwickshire” (Ph.D. dissertation, University of Warwick, 1983) is one of the few works to explore both prison and asylum and movements of inmates between the two institutions.

25 See Roy Porter, *A Social History of Madness: Stories of the Insane* (London: Weidenfeld and Nicholson, 1987), 25. See also Porter’s call to explore patient’s testimonies: “The Patient’s View: Doing Medical History From Below,” *Theory and Society* 14 (1985): 175-198. For responses to Porter’s call, see Flurin Condrau, “The Patient’s View Meets the Clinical Gaze,” *Social History of Medicine* 20 (2017): 525-540 and Alexandra Bacopoulos-Viau and Aude Fauvel, “The Patient’s Turn: Roy Porter and Psychiatry’s Tales, Thirty Years On,” *Medical History* 60 (2016): 1-18, and the essays included in their special issue “Tales from the Asylum. Patient Narratives and the (De)construction of Psychiatry.”

26 See Andrew Scull, *Madness in Civilization* (London: Thames & Hudson, 2015), 139-144 and Peter McCandless, “Liberty and Lunacy: The Victorians and Wrongful Confinement,” in *Madhouses, Mad-Doctors, and Madmen*, ed. Andrew Scull (London: Athlone, 1981), 339-362, for accounts of wrongful confinement in asylums.

27 Michel Foucault, “Intellectuals and Power,” in *Language, Counter-Memory, Practice*, ed. D.F. Bouchard (Ithaca, NY: Cornell University Press, 1977), 205-217; idem, *Discipline and Punish: The Birth of the Prison*, translated from the French by Alan Sheridan (London: Allen Lane, 1977); Michael Ignatieff, *A Just Measure of Pain: The Penitentiary in the Industrial Revolution 1750-1850* (London: Macmillan, 1978); David Garland, *Punishment and Modern Society* (Oxford: Clarendon, 1990). Ignatieff asserted an interest in prisoners’ resistance, though, as Anderson and Pratt suggest, his work provides little evidence of this: Anderson and Pratt, “Prisoner Memoirs,” 179.

28 Brown and Clare, “History of Experience,” 69, 68.

29 For a detailed account of the regime at Pentonville and its impact on the minds of its inmates, see Catherine Cox and Hilary Marland, “‘He Must Die or Go Mad in This Place’: Prisoners, Insanity and the Pentonville Model Prison Experiment, 1842-1852,” *Bulletin of the History of Medicine* 92 (2018): 78-109.

30 House of Commons Papers, Second Report of the Commissioners for the Government of Pentonville Prison, 1844 [536], 18-20, Appendix B, No. 3 “Routine of a Day,” 190.

31 Scull, *Most Solitary*, 109; Ignatieff, *Just Measure*, 213.

32 Randall McGowen, “The Well-Ordered Prison. England, 1780-1865,” in *The Oxford History of the Prison*, ed. Norval Morris and David J. Rothman (Oxford: Oxford University Press, 1995), 79-109, 101. See also Robin Evans, *The Fabrication of Virtue: English Prison Architecture, 1750–1840* (Cambridge: Cambridge University Press, 1982), 318-345.

33 J. Jebb, *Modern Prisons: Their Construction and Ventilation* (London: John Weale, 1844), 6, 9-10; Clive Emsley, “Jebb, Sir Joshua (1793–1863)” (23 September 2004). *DNB*.

34 Jebb, *Modern Prisons*, 10.

35 See e.g. Forsythe, *Reform*, ch. 2.

36 Roy Porter, “Hearing the Mad: Communication and Excommunication,” in *Proceedings of the 1^st^ European Congress on the History of Psychiatry and Mental Health Care*, ed. Leonie de Goei and Joost Vijselaar (Rotterdam: Erasmus Publishing, 1993), 338-352, 338.

37 Grass, *Self*, 31-32.

38 Ibid., 31.

39
*The Prison Chaplain: A Memoir of the Rev. John Clay, B.D., Late Chaplain of the Preston Gaol*, by His Son, The Rev. Walter Lowe Clay, M.A. (Cambridge and London: Macmillan, 1861), 207; see also Ignatieff, *Just Measure*, 196-198. For prisoner narratives in the US in the early years of the penitentiary, see Jennifer Graber, “Engaging the Trope of Redemptive Suffering: Inmate Voices in the Antebellum Prison Debates,” *Pennsylvania History: A Journal of Mid-Atlantic Studies* 79 (2012): 209-233; Jodi Schorb, *Reading Prisoners: Literature, Literacy, and the Transformation of American Punishment, 1700-1845* (New Brunswick, NJ: Rutgers University Press, 2014).

40 Berkshire Record Office, Berkshire Sessions Order Books, Q/SO 22 January 1849-July 1850, 15 October 1849, Reading Gaol: Chaplain’s Report, 198; John Field, *Prison Discipline: And the Advantages of the Separate System of Imprisonment, with a Detailed Account of the Discipline Now Pursued in the New County Gaol at Reading*, 2 vols (London: Longman, Brown, Green and Longmans, 1848), vol. 1, 347. See also Rosalind Crone, “The Great ‘Reading’ Experiment: An Examination of the Role of Education in the Nineteenth-Century Gaol,” *Crime, History & Societies* 16 (2012): 47-74.

41 Third Report of the Commissioners for the Government of Pentonville Prison, 1845 [613], 13, 11.

42 Forsythe, *Reform*, 48; Kingsmill reputedly made some 100,000 cell visits over the course of his career: B. Forsythe, “Kingsmill, Joseph (1805/6–1865)” (23 September 2004), *DNB*.

43 Joseph Kingsmill, *Chapters on Prisons and Prisoners*, 3^rd^ edition (London: Longman, Brown, Green and Longmans, 1854), 218.

44 Charles B. Gibson, *Life Among Convicts*, 2 vols (London: Hurst and Blackett, 1863), vol. 1, 91, 100.

45 The National Archives (TNA) PCOM 2/353 Pentonville Prison, Middlesex: Chaplain’s Journal, 1846-51.

46 Ignatieff, *Just Measure*, 199.

47 Henry Mayhew and John Binny, *The Criminal Prisons of London and Scenes from Prison Life* (London: Griffin, Bohn & Co., 1862), 115.

48 “Letter to the Editor,” *The Times*, 11 January 1847.

49 U.R.Q. Henriques, “The Rise and Decline of the Separate System of Prison Discipline,” *Past and Present* 54 (1972): 61-93, 86.

50 Report on the Discipline and Management of the Convict Prisons (RDMCP), 1852, 1852-53 [1659], Appendix, “Extracts from the Report of the Chaplain of Pentonville Prison for 1852,” 93-94.

51 TNA PCOM 2/353 Chaplain’s Journal, 1846-51, 13 February 1849, 143.

52 Wakefield Record Office, QS 10/56, Quarter Sessions Order Book, October 1846-April 1850, Wakefield Adjourned Sessions, 9 December 1847, 14 December 1848, Surgeons Reports.

53 RDMCP, 1852, 1852-53 [1659], Appendix, “Report of Dr. Baly on the Effects of Separate Confinement,” 94.

54 For the drive for uniformity, see Miles Ogborn, “Discipline, Government and the Law: Separate Confinement in the Prisons of England and Wales, 1830-1877,” *Transactions of the Institute of British Geographers* 20 (1995): 295-311.

55 Seán McConville, *English Local Prisons 1860-1900: Next Only to Death* (London: Routledge, 1995). For the experiences of Liverpool Borough Gaol, see Catherine Cox and Hilary Marland, “‘Unfit for Reform or Punishment’: Mental Disorder and Discipline in Liverpool Borough Gaol in the Late Nineteenth Century,” forthcoming *Social History* (2019).

56 For penal conditions and regimes after the 1860s and 1870s, see Forsythe, *Reform*, chs 6, 8.; idem, “Centralisation and Local Autonomy: The Experience of English Prisons 1820-1877,” *Journal of Historical Sociology* 4 (1991): 317-345; McConville, *English Local Prisons*.

57 Forsythe, “Loneliness.”

58 Edwin Pears, *Prisons and Reformatories at Home and Abroad, being the Transactions of the International Penitentiary Congress held in London, July 3-13, 1872* (London: Longman’s, Green and Co., 1872), 305. Cited Cox and Marland, “Broken Minds,” 19.

59 McConville, *English Local Prisons*, 290; William A. Guy, “On Insanity and Crime; and On The Plea of Insanity in Criminal Cases,” *Journal of the Statistical Society of London* 32 (1869): 159-191, 186.

60 Anderson and Pratt, “Prisoner Memoirs,” 180.

61 Frederick Brocklehurst, *I was in Prison* (London: T. Fisher Unwin, 1898).

62 John Hay, *A Gross Miscarriage of Justice. Seven Years of Penal Servitude or the Value of a Royal Pardon* (London: The Literary Revision Society, 1894), 32. Cited Priestley, *Victorian Prison Lives*, 128.

63 Balfour, *My Prison Life*.

64 “Five Years’ Penal Servitude,” *The Saturday Review*, 17 November 1877, 623-624, 623. See also *The Academy*, 22 December 1877, 567; *The Athenaeum*, 17 November 1877, 624-625.

65 Schwan, *Convict Voices*, 94.

66 On Wilde, see Stoneley, “Looking at the Others.”

67 Balfour, *My Prison Life*, x-xi.

68 B.2.15, *Broad-Arrow Men*, 129.

69 Constance Lytton, *Prisons and Prisoners: Some Personal Experiences by Constance Lytton and Jane Wharton, Spinster* (London: Heinemann, 1914); Sarah Richardson “Suffragette Diary,” https://histprisonhealth.com/2018/02/02/suffragette-diary/; LSE Library (Archives and Special Collections, LSE Library, KELLEY/2/16).

70 While women comprised only a small proportion of the prison population (some seventeen per cent in the 1890s according to Zedner), Susan Willis Fletcher and Florence Elizabeth Maybrick produced expansive memoirs of their experiences that prefaced the better-known writings of suffragette prisoners. Lucia Zedner, *Women, Crime, and Custody in Victorian England* (Oxford: Clarendon, 1991), 1; Fletcher, *Twelve Months*; Florence Elizabeth Maybrick, *Mrs. Maybrick’s Own Story. My Fifteen Lost Years* (New York: Funk & Wagnalls, 1905), 110. See also Schwan, *Convict Voices*, chs 4 and 6.

71 Fletcher, *Twelve Months*, 319.

72 Schwan, *Convict Voices*, 91, 116-119.

73 Fletcher, *Twelve Months*, 350.

74 Schwan, *Convict Voices*, 118.

75 Maybrick, *Mrs. Maybrick’s Own Story*, 64.

76 Anon., “Twenty Years’ Penal Servitude,” *The Chambers’s Journal*, 26 October 1867, 673-678, 677.

77 B.2.15, *Broad-Arrow Men*, 64.

78 Schwan, *Convict Voices*, 134.

79 Maybrick, *Mrs. Maybrick’s Own Story*, 67.

80 Ibid., 68, 74.

81 Ibid., 81.

82 Brocklehurst, *I was in Prison*, xvii, 120.

83 Ibid., 6.

84 Austin Bidwell, *From Wall Street to Newgate* (London: Forum Press, 1895), 397.

85 Fletcher, *Twelve Months*, 328.

86 “An Observation Cell,” cited as an Appendix to ch. 18 in Stephen Hobhouse and A. Fenner Brockway, *English Prisons Today: Being the Report of the Prison System Enquiry Committee* (London: Longmans, Green and Co., 1922), 293.

87 W.B.N., *Penal Servitude* (London: William Heinemann, 1903), 30-31.

88 Balfour, *My Prison Life*, 43, 48.

89 Ibid., 122.

90
*Her Majesty’s Prisons: Their Effects and Defects*, By one who has tried them, vols 1 and 2 (London: Sampson Low, Marsten, Searle & Rivington, 1881), vol. 1, 53, 263-264.

91 Scott, *The Human Side*, 13.

92 Ibid., 139.

93 Fletcher, *Twelve Months*, 353-354; Anon., *Pentonville Prison from Within (with an Actress in the Background)* (With a preface by Folliott Stokes) (London: Greening & Co., 1904), 69-70; *Her Majesty’s Prisons*, vol. 2, 128-129, 252.

94
*Her Majesty’s Prisons*, vol. 2, 252-255.

95 Anon., *Pentonville Prison*, 69, 87, 89.

96 McConville, *English Local Prisons*, 298-303.

97
*Her Majesty’s Prisons*, vol. 2, 58, 93; *Five Years' Penal Servitude*, by One Who has Endured It (London: Richard Bentley & Son, 1878), 96.

98 Maybrick, *Mrs Maybrick’s Own Story*, 178, 172-173.

99 Michael Davitt, *The Prison Life of Michael Davitt* (Dublin: Lalor, 1886), 11.

100 Michael Davitt, *Leaves from a Prison Diary; Or, Lectures to a “Solitary” Audience* (London: Chapman and Hall, 1885), reprinted with introduction by T.W. Moody (Shannon: Irish University Press, 1972), vol. 1, 142-146. See also Sean T. O’Brien, “The Prison Writing of Michael Davitt,” *New Hibernia Review* 14 (2010): 16-32.

101 W.B.N., *Penal Servitude*, 148.

102 Ibid., 150.

103 B.2.15, *Broad-Arrow Men*, 163-164.

104
*Towards Tomorrow: The Autobiography of Fenner Brockway* (London: Hart-Davis, MacGibbon, 1977), 52.

105 E. Williamson Mason, *Made Free in Prison* (London: George Allen & Unwin, 1918), 134.

106 Ibid.

107 Davitt, *Leaves from a Prison Diary*, 171-172.

108 B.2.15, *Broad-Arrow Men*, 64.

109 W.B.N., *Penal Servitude*, 26.

110 Anon., *Pentonville Prison*, 52-54.

111 To the Editor of the Daily Chronicle, 23 March [1898], 195.

112
*Convict Life; or, Revelations Concerning Convicts and Convict Prisons*, by a Ticket-of-Leave Man (London: Wyman & Sons, 1879), 184.

113
*Five Years' Penal Servitude*, 53.

114 Jeremiah O’Donovan Rossa, *Irish Rebels in English Prisons* (New York: P.J. Kenedy, 1899), 119.

115 Ibid., 105.

116 Balfour, *My Prison Life*, 48.

117 B.2.15, *Broad-Arrow Men*, 87.

118 Fletcher, *Twelve Months*, 331.

119 Stuart Wood, *Shades of the Prison House: A Personal Memoir* (London: Williams & Norgate, 1932), 32. See also Balfour, *My Prison Life*, 49-50.

120 Fletcher, *Twelve Months*, 403.

121 Maybrick, *Mrs Maybrick’s Own Story*, 192, 80, 105.

122 Fletcher, *Twelve Months*, 404. Cited Zedner, *Women*, 140.

123 W.B.N., *Penal Servitude*, 31.

124 Brocklehurst, *I was in Prison*, 17.

125 Hobhouse and Brockway, *English Prisons*, 571.

126 Stephen Hobhouse, *Forty Years and An Epilogue. An Autobiography (1881-1951)* (London: James Clarke & Co., 1951), 165-166.

127 Hobhouse, “An English Prison from Within,” 33-34.

128 Erving Goffman, *Asylums: Essays on the Social Situation of Mental Patients and Other Inmates* (New York: Doubleday, 1961); Hobhouse and Brockway, *English Prisons*, 506-513. See also Brown, *English Society*, 21-22.

129 Balfour, *My Prison Life*, 215-216; Lauterbach, “‘From the Slums.’”

130
*Five Years' Penal Servitude*, 65.

131 O’Donovan Rossa, *Irish Rebels*, 91.

132 Stephen Hobhouse, “An English Prison from Within,” *The Quarterly Review* 230 (July 1918): 21-37, 21.

133 Schwan, *Convict Voices*, 138.

134 Ibid., 117.

135 Fletcher, *Twelve Months*, 323, 337-338.

136 Maybrick, *Mrs Maybrick’s Own Story*, 81, 204.

137 Ibid., 83, 81.

138 Review of *Five Years’ Penal Servitude*, *The Academy*; Anderson and Pratt, “Prisoner Memoirs,” 192-193.

139 Royal Commission into Penal Servitude Acts, Minutes of Evidence (Kimberley Commission), 1878-79, XXXVII, Evidence of Michael Davitt, 20 June 1878, 515-544.

140 W.J. Forsythe, *Penal Discipline, Reformatory Projects and the English Prison Commission 1895-1939* (Exeter: University of Exeter Press, 1990), 19-30; Victor Bailey, “English Prisons, Penal Culture, and the Abatement of Imprisonment, 1895-1922,” *Journal of British Studies* 36 (1997): 285-324.

141 Charles Reade’s novel, *It is Never too Late to Mend* (1856), later produced as a play, attacked prison abuses, while John Galsworthy corresponded directly with Prison Commissioner, Sir Evelyn Ruggles-Brise and Home Secretary, Winston Churchill in protesting against the continued use of solitary confinement, voicing his criticisms in *The Nation* and *Daily News* and in his play ‘Justice’ (1910).

142 Anon., “Prison Reform,” *The Speaker*, 27 April 1895, 452-453, 453.

143 TNA, HO 45/13658, Prisons and Prisoners: Separate or cellular confinement: general papers, 1909-1930, Solitary Confinement: An Open Letter to the Home Secretary (Gladstone) By John Galsworthy, 8 May 1909, Reproduced in *The Nation*, 8 May 1909, 7.

144
*The Nation*, 8 May 1909, 8-9.

145 Forsythe, *Penal Discipline*, 26.

146 Report from the Departmental Committee on Prisons, Minutes of Evidence (Gladstone Committee), 1895, LVI, Evidence of Mr. E., 7 December 1894, 9737-9744.

147 See e.g. John Baker, “Insanity in English Local Prisons, 1894-95,” *Journal of Mental Science* 42 (1896): 294-302 for claims that the number of insane in prisons was exaggerated.

148 William Douglas Morrison, “Are our Prisons a Failure?” *The Fortnightly Review* 61 (April 1894): 459-469, 468.

149 Wilde, “Ballad of Reading Gaol,” 570.

150 B.2.15, *Broad-Arrow Men*, 184, 187. As a recent report of the English Prison Reform Trust highlights, the “toxic” effects of segregation are still evident in prisons today, with people in prison reporting on the anxiety, insomnia, depression, and difficulty concentrating resulting from solitary confinement: Sharon Shalev and Kimmett Edgar, *Deep Custody: Segregation Units and Close Supervision Centres in England and Wales* (London: Conquest Litho, 2015).

